# Electrochemical Biosensors for Exosome Detection: Current Advances, Challenges, and Prospects for Glaucoma Diagnosis

**DOI:** 10.3390/s26020433

**Published:** 2026-01-09

**Authors:** María Moreno-Guzmán, Juan Pablo Hervás-Pérez, Laura Martín-Carbajo, María José Crespo Carballés, Marta Sánchez-Paniagua

**Affiliations:** 1 Department of Chemistry in Pharmaceutical Sciences, Faculty of Pharmacy, Complutense University of Madrid, 28040 Madrid, Spain; marimore@ucm.es (M.M.-G.); jphervas@ucm.es (J.P.H.-P.); lmartinc@ucm.es (L.M.-C.); 2 Opthalmology Service, Hospital Universitario Infanta Leonor, 28031 Madrid, Spain; macres02@ucm.es

**Keywords:** exosomes, electrochemical biosensors, glaucoma, biomarkers, diagnosis

## Abstract

**Highlights:**

- Electrochemical biosensors enable highly sensitive, low-volume detection of exosomal surface markers and intravesicular cargos in biological fluids.
- Recent advances in nanomaterials, microfluidics, and signal amplification enhance sensitivity, multiplexing capability, and translational potential.
- These platforms show strong potential for early glaucoma diagnosis through the analysis of aqueous humor–derived exosomes.
- The development of miniaturized point-of-care biosensors may allow noninvasive monitoring of glaucoma-associated molecular biomarkers.

**Abstract:**

Glaucoma is a leading cause of irreversible blindness worldwide, with its asymptomatic progression highlighting the urgent need for early, minimally invasive biomarkers. Exosomes derived from the aqueous humor (AH) have emerged as promising candidates, as they carry proteins, nucleic acids, and lipids that reflect the physiological and pathological state of ocular tissues such as the trabecular meshwork and ciliary body. However, their low abundance, nanoscale size, and the limited volume of AH complicate detection and characterization. Conventional methods, including Western blotting, PCR or mass spectrometry, are labor-intensive, time-consuming, and often incompatible with microliter-scale samples. Electrochemical biosensors offer a highly sensitive, rapid, and low-volume alternative, enabling the detection of exosomal surface markers and internal cargos such as microRNAs, proteins, and lipids. Recent advances in nanomaterial-enhanced electrodes, microfluidic integration, enzyme- and nanozyme-mediated signal amplification, and ratiometric detection strategies have significantly improved sensitivity, selectivity, and multiplexing capabilities. While most studies focus on blood or serum, these platforms hold great potential for AH-derived exosome analysis, supporting early-stage glaucoma diagnosis, monitoring of disease progression, and evaluation of therapeutic responses. Continued development of miniaturized, point-of-care electrochemical biosensors could facilitate clinically viable, noninvasive exosome-based diagnostics for glaucoma.

## 1. Context of the Study

Glaucoma remains one of the leading causes of irreversible blindness worldwide, and its asymptomatic progression underscores the critical need for early and reliable biomarkers capable of detecting disease activity before significant visual damage occurs. Among emerging molecular sources, exosomes derived from the aqueous humor have gained increasing attention due to their ability to transport disease-related proteins, nucleic acids, and signaling molecules released by the trabecular meshwork, ciliary body, and other anterior segment tissues. These nanoscale vesicles offer a unique and minimally invasive window into the molecular events driving intraocular pressure dysregulation and optic nerve damage. However, the detection and characterization of aqueous humor-derived exosomes remain technically challenging because of their low abundance, small size, and the limited volume of aqueous humor obtainable in clinical settings.

Conventional analytical techniques for studying exosomes can be broadly classified into methods that detect the vesicles directly and those that analyze their molecular cargo. Approaches such as ultracentrifugation, nanoparticle tracking analysis, transmission electron microscopy, and high-resolution flow cytometry focus on the isolation, visualization, or quantification of intact exosomes, providing information about particle concentration, size distribution, and morphology. In contrast, techniques including Western blotting, ELISA, RT-qPCR, and mass spectrometry target exosomal biomarkers. While direct detection methods characterize the physical presence of exosomes, biomarker-oriented methods reveal their functional and molecular content. However, these conventional approaches are often limited by labor-intensive workflows, long processing times, and poor compatibility with the extremely small volumes of aqueous humor available in clinical settings. Electrochemical biosensors have emerged as a promising alternative, offering high sensitivity, low sample requirements, and the potential for miniaturization and real-time analysis.

Within this context, the present review provides an overview of glaucoma pathology, the role of exosomes in ocular physiology, and the biomarker potential of the biomolecular profile of AH-derived exosomes, the limitations of conventional analytical techniques and the potential use of electrochemical biosensing platforms. By examining the biochemical characteristics of AH-derived exosomes, evaluating sensor design strategies, and analyzing their diagnostic relevance, this review aims to consolidate current knowledge on electrochemical biosensors for exosome detection in glaucoma.

## 2. Glaucoma: Clinical and Pathophysiological Significance

Glaucoma is a complex, heterogeneous group of optic neuropathies characterized by the progressive, irreversible loss of retinal ganglion cells (RGCs) and their axons, leading to visual field defects and, ultimately, blindness if untreated. While elevated intraocular pressure (IOP) remains the most significant and modifiable risk factor—and is the primary target of current therapies—glaucoma pathogenesis extends well beyond pressure-mediated damage [1,2,3].

Epidemiologically, glaucoma imposes a growing global health burden. It is one of the most common neurodegenerative diseases and the leading cause of irreversible blindness, affecting ~80 million people worldwide and is predicted to increase to 112 million by 2040 [1,4,5,6]. Although the prevalence in adults aged 40–80 is estimated at around 3.5%, projections suggest that tens of millions more people will be affected in the coming decades [7]. In Europe, for example, more than half of identified glaucoma cases remain undiagnosed, and prevalence is expected to rise further [8]. Mechanistically, the disease involves a multifactorial cascade. Elevated IOP can cause mechanical stress on the lamina cribrosa, impairing axonal transport in RGCs and triggering neurodegeneration [8]. In addition to this, oxidative stress plays a key role: reactive oxygen species (ROS) accumulation, mitochondrial dysfunction, and decreased antioxidant defenses contribute both to trabecular meshwork dysfunction and to RGC death [9,10,11]. Neuroinflammation is another central feature: glial cells (microglia, astrocytes, and Müller cells) become reactive, secreting pro-inflammatory cytokines, disrupting the blood-retinal barrier, and exacerbating neurodegeneration [12,13,14,15].

In this context, apoptosis has long been recognized as the predominant mode of RGC loss in glaucoma. Both intrinsic and extrinsic apoptotic pathways—characterized by mitochondrial cytochrome-c release and caspase-3/9 activation—have been demonstrated in experimental models of ocular hypertension and in human glaucomatous retinas [16,17]. Apoptotic processes are also evident in trabecular meshwork cells, where they contribute to outflow pathway dysfunction and sustain IOP elevation [18]. Increasingly, however, apoptosis is viewed not as an isolated mechanism but as part of a broader spectrum of regulated cell-death programs that operate in glaucomatous neurodegeneration, forming a continuum that includes ferroptosis, necroptosis, and autophagy-related pathways.

Recent research has also identified novel molecular pathways in glaucoma. For example, ferroptosis—an iron-dependent form of regulated cell death driven by lipid peroxidation—has emerged as a promising therapeutic target, linking iron dysregulation, oxidative stress, and RGC neurodegeneration [16,17].

Moreover, at the level of the optic nerve head, astrocytes show region-specific, temporally distinct reactive responses to elevated IOP, suggesting that different subpopulations of glial cells may contribute differentially to glaucomatous damage depending on disease stage [19,20,21].

Given that many patients continue to progress despite well-controlled IOP, there is an urgent need for neuroprotective and non-IOP–lowering therapies. Current research is exploring gene therapy, stem-cell approaches, vascular modulation, and anti-inflammatory treatments to preserve or restore RGCs. In summary, glaucoma is not merely an “eye-pressure disease”: it is a multifaceted neurodegenerative disorder involving oxidative damage, inflammation, metabolic dysfunction, and mechanical stress. Understanding these diverse mechanisms is essential for developing next-generation therapies that go beyond intraocular pressure control [22].

## 3. Aqueous Humor Exosomes in Glaucoma: Biological Roles, Diagnostic Challenges and Clinical Prospects

Aqueous humor (AH) is a dynamic intraocular fluid essential for maintaining the metabolic balance of anterior segment tissues and regulating intraocular pressure. In recent years, extracellular vesicles, particularly exosomes, have been recognized as critical molecular components of AH, acting as central mediators of intercellular communication in both physiological and pathological ocular processes [23,24]. These nanosized vesicles, typically 30–150 nm in diameter, are enclosed by a phospholipid bilayer that preserves their bioactive cargo from enzymatic degradation and enables selective interactions with recipient cells [24,25]. Exosomes contain conserved signature proteins as well as cell-type–specific markers, which allow the identification of their cellular origin. Initially considered a mechanism for cellular waste disposal, they are now understood to be active mediators of intercellular communication, transporting proteins, lipids, mRNA, microRNAs, transcription factors, and other small RNAs that modulate the behavior of neighboring or distant cells [26,27,28].

In the eye, exosomes present in AH are derived from a variety of ocular and systemic cell sources, including trabecular meshwork cells, epithelial cells, and bone-marrow–derived cells [23]. Their presence across multiple biological fluids—such as blood, urine, tears, vitreous humor, and AH—highlights their systemic biological relevance [29]. Their small size and lipid composition also facilitate their passage across biological barriers, including the blood–retinal barrier, enabling both local and long-range molecular transport. As emphasized by Beit-Yannai (2025), the specialized molecular cargo and cell-specific markers of exosomes position them as potent regulators of ocular homeostasis and disease mechanisms [24]. In the context of glaucoma, alterations in exosomal composition and intercellular signaling may reflect pathological dysregulation within the anterior segment, supporting their exploration as biomarkers for disease detection and progression monitoring [24,30,31,32,33].

Despite their biological and diagnostic potential, the clinical translation of AH-derived exosomes faces significant analytical and methodological challenges. The collection of AH and other ocular biofluids typically yields only microliter volumes, obtainable during interventions such as anterior chamber paracentesis or cataract surgery, resulting in extremely low extracellular vesicle (EV) concentrations that hinder recovery and downstream analysis [33,34]. The small size, structural heterogeneity, and overlapping biochemical signatures of exosomes with other EV subtypes further complicate their reliable isolation and characterization [24,35]. Current isolation techniques—including differential ultracentrifugation, size-exclusion chromatography, and immunoaffinity capture—entail trade-offs among purity, yield, and reproducibility, which become particularly pronounced when working with the limited sample volumes available from ocular fluids [34,35]. Additionally, co-isolated contaminants such as lipoproteins or non-exosome vesicles can compromise diagnostic accuracy and interfere with functional assays [34]. Methodological inconsistencies across studies, including variability in EV quantification, characterization, and reporting practices, further limit the reproducibility and comparability of results [33].

Disease-specific modifications in exosome abundance, cargo composition, and cellular origin have been documented in glaucoma and other ocular diseases, including diabetic retinopathy, keratoconus, and uveitis. These alterations underscore their promise as biomarkers while simultaneously highlighting the necessity for sensitive, standardized detection methodologies capable of resolving disease-dependent exosomal signatures [35]. Understanding the physiological and pathological roles of ocular exosomes is therefore essential for accurately interpreting biomarker-derived signals, as emphasized by Beit-Yannai (2025) [24].

Beyond diagnostics, exosomes also present significant therapeutic opportunities. Engineered or stem–cell-derived exosomes can serve as delivery vehicles for proteins, nucleic acids, and small molecules, offering innovative strategies for tissue regeneration, immune modulation, and targeted drug delivery to ocular tissues [34,35]. However, the clinical application of therapeutic EVs remains limited by challenges related to targeting specificity, reproducibility of exosome production, safety concerns, and evolving regulatory frameworks, as noted by Su et al. (2024) [34].

Collectively, advances in the understanding of AH-derived exosomes are reshaping current perspectives on glaucoma pathophysiology and opening new avenues for the development of diagnostic and therapeutic tools. Continued progress will depend on the optimization and standardization of isolation technologies, the development of highly sensitive detection platforms, and a deeper elucidation of the mechanistic roles of exosomes in ocular homeostasis and disease.

## 4. Analytical Approaches for Exosome Detection

### 4.1. Conventional Methods

Conventional analytical approaches for studying exosomes can be broadly classified into techniques aimed at detecting intact vesicles and methods focused on analyzing their molecular constituents [36]. Techniques targeting whole particles—such as ultracentrifugation, nanoparticle tracking analysis (NTA), transmission electron microscopy (TEM), and flow cytometry—provide measurements of concentration, size distribution, and morphological features. In contrast, molecular analysis tools, including Western blotting, ELISA, RT-qPCR, and mass spectrometry, are used to characterize exosome-associated proteins, lipids, and nucleic acids, offering insights into their molecular identity and biological function.

Despite their widespread use, these techniques face significant limitations when applied to aqueous humor (AH), where only microliter-scale volumes can be collected during clinical procedures. Ultracentrifugation, the most commonly employed isolation strategy, is labor-intensive, time-consuming, and often yields insufficient recovery for downstream analysis, making it poorly suited to low-input ocular samples [37]. As described by some authors classical isolation methods do not meet modern clinical demands for high yield, reproducibility, and scalability [38]. Although optimized protocols have been established for plasma and cell culture supernatants, their applicability to AH remains uncertain due to the extremely limited sample volumes and the reproducibility challenges inherent to ocular biofluids [38].

Characterization methods present additional constraints. The heterogeneity of extracellular vesicle (EV) populations, co-isolation of contaminants, and variations in vesicle concentration all complicate qualitative and quantitative analysis [39].

Nanoparticle tracking analysis (NTA), while widely used for size and particle quantification, is susceptible to dilution-dependent bias and cannot discriminate between exosomes and similarly sized entities such as protein aggregates or lipoproteins [40]. Moreover, reliable NTA measurements typically require particle concentrations around 10^8^ particles/mL—levels rarely achieved in AH samples.

Flow cytometry faces analogous difficulties: conventional cytometers lack the sensitivity to detect particles <200 nm, and even high-resolution platforms are affected by “swarm effects,” wherein multiple vesicles are erroneously counted as single events, reducing analytical accuracy [41].

Techniques for analyzing exosome-derived molecular constituents, including Western blotting, ELISA, PCR, and mass spectrometry, are commonly employed to detect proteins and nucleic acids. Western blot and ELISA are considered gold standards for protein analysis; however, they require relatively large sample volumes, involve lengthy processing times, and exhibit limited sensitivity, restricting their applicability to low-volume fluids such as aqueous humor [36,42]. PCR, widely used to detect exosomal nucleic acids such as miRNAs and mRNAs, offers high sensitivity and specificity but necessitates careful sample preparation and can be inhibited by components present in biological fluids [43,44]. Mass spectrometry enables high-resolution molecular profiling and multiplexed detection of proteins, yet it requires extensive sample preparation, specialized instrumentation, and costly reagents, making it impractical for routine clinical or point-of-care use [45,46]. Additionally, variations in EV isolation protocols, sample handling, and the presence of contaminants across different biofluids can further impact the reproducibility and reliability of these analyses [42].

Collectively, these limitations underscore the need for analytical platforms capable of high sensitivity, minimal sample consumption, standardized workflows, and rapid processing, particularly for clinical applications in glaucoma, where early diagnosis and longitudinal monitoring depend on the ability to robustly profile exosomal biomarkers in ultra-low-volume ocular samples.

### 4.2. Electrochemical Biosensors

Electrochemical biosensors represent a highly promising platform for the sensitive, rapid, and miniaturizable detection of exosomes and their molecular cargo [31]. These devices can be engineered to specifically target exosomal surface proteins (e.g., CD9, CD63, CD81, EpCAM), nucleic acids (such as microRNAs or long non-coding RNAs), or enzymatic activities, converting biological recognition events into measurable electrical signals via voltammetric, amperometric, or impedimetric readouts [42]. Recent advancements in nanomaterials and surface functionalization have significantly enhanced sensor performance by increasing surface area, improving conductivity, and providing abundant sites for aptamer or antibody immobilization, enabling ultrasensitive detection limits [46,47,48].

Integration with microfluidic systems reduces sample volume requirements, accelerates analysis times, and facilitates point-of-care deployment, which is particularly advantageous for ophthalmic diagnostics where only microliters of aqueous humor can be safely collected [31,36]. Sophisticated sensing architectures—including aptamer-based sensors, immunosensors, impedance-based detection systems, catalytic amplification platforms, and lab-on-a-chip devices—enhance signal transduction efficiency and expand analytical capabilities [31,47]. Applied to exosome detection, these innovations hold significant potential for early disease diagnosis, real-time monitoring of therapeutic responses, and personalized treatment strategies.

Overall, electrochemical biosensors provide a rapid, label-free, low-volume, and clinically relevant approach for profiling exosome-derived biomarkers, overcoming many limitations of conventional analytical techniques while maintaining high sensitivity, low cost, and compatibility with miniaturized or portable formats [46,48].

Despite these promising developments, research specifically focusing on aqueous humor-derived exosomes remains relatively limited. Most current exosome biosensing studies concentrate on blood, urine, or saliva, leaving a critical gap in ophthalmology—a field where molecular diagnostics could substantially improve patient outcomes through earlier intervention [49,50].

## 5. Targets in Aqueous Humor–Derived Exosomes

Exosomes present in the aqueous humor (AH) carry a diverse array of molecular features that can serve as potential biomarkers for glaucoma. Their surface proteins mediate cell recognition, signaling, and intercellular communication, while internal cargos, including enzymes, regulatory proteins, and cytokines, reflect the functional state of their parent cells [25,30]. Comprehensive profiling of both surface and internal exosomal components provides valuable insights into pathological alterations in ocular tissues, such as trabecular meshwork remodeling, ciliary body dysfunction, and corneal endothelial stress [42]. Recent proteomic and cytokine analyses have identified specific molecules, including GAS6 and SPP1, as promising glaucoma markers, highlighting the potential of AH-derived exosomes for diagnostic, prognostic, and therapeutic monitoring strategies [30,42].

### 5.1. Exosomal Surface Proteins in Aqueous Humor as Glaucoma Biomarkers

Exosomal surface proteins are among the most accessible and widely utilized biomarkers for vesicle identification and disease profiling. These markers not only enable the detection and quantification of exosomes but also provide insights into their cellular origin and potential pathological relevance. Classical tetraspanins—CD9, CD63, and CD81—serve as universal exosome markers, facilitating vesicle capture and characterization. In addition, disease- or tissue-specific surface proteins, including integrins, MHC molecules, fibronectin, and ICAM-1, offer pathological specificity and indicate the cellular source of the exosomes [30,42]. In the context of glaucoma, surface proteins on aqueous humor-derived exosomes are particularly informative. They reflect cellular stress or pathological alterations in ocular tissues such as the trabecular meshwork, ciliary body, corneal endothelium, and infiltrating immune cells within the anterior chamber. Clinical studies have shown that exosomal tetraspanin levels are elevated in glaucoma patients; for instance, total CD63, captured CD63, total CD81, and captured CD9 concentrations were significantly higher in the aqueous humor of individuals with primary open-angle glaucoma (POAG) compared to controls [30]. The accessibility and abundance of these surface markers make them ideal targets for biosensor-based detection platforms, enabling selective capture, quantitative analysis, and monitoring of disease progression or therapeutic responses [42,51].

### 5.2. Internal Exosomal Cargos and Their Diagnostic Relevance

Exosomal internal components, also referred to as molecular cargo, comprise a diverse set of molecules enclosed within the vesicle and transported between cells. These include nucleic acids (e.g., microRNAs, long non-coding RNAs, and mRNAs), proteins (including signaling molecules, enzymes involved in apoptosis, and extracellular matrix remodeling), lipids, and small metabolites [30,42]. These molecules reflect the physiological or pathological state of the parent cell and provide critical insights into disease mechanisms [25]. In glaucoma, the analysis of aqueous humor-derived exosomal internal components can reveal early molecular alterations in ocular tissues such as the trabecular meshwork, ciliary body, and corneal endothelium, providing opportunities for diagnostic and therapeutic monitoring [30,42].

#### 5.2.1. Exosomal microRNAs (miRNAs)

Exosomal microRNAs are small, non-coding RNAs that regulate gene expression post-transcriptionally and are remarkably stable within exosomes [52]. In the aqueous humor of glaucoma patients, these vesicle-encapsulated miRNAs are highly disease-specific and serve as sensitive biomarkers, providing insights into early molecular alterations before clinical symptoms appear. Exosomal miRNAs regulate key pathways involved in glaucoma pathophysiology, including inflammation (e.g., miR-146a, miR-155), fibrosis and extracellular matrix remodeling (e.g., miR-21, miR-29 family), angiogenesis (e.g., miR-126, miR-200b), and oxidative stress (e.g., miR-34a). Several studies have identified glaucoma-specific miRNA signatures; for instance, miR-21-5p, miR-92b-3p, miR-99a-5p, miR-486-3p, miR-486-5p, and miR-1260a constitute a glaucoma-associated profile, while miR-486-5p, miR-204, and miR-184 were found abundantly in multiple patient samples [23]. Recent studies have identified distinct miRNA profiles in the aqueous humor of glaucoma patients, highlighting their potential as biomarkers and therapeutic targets. MicroRNAs transported within exosomes are remarkably stable, disease-specific, and highly informative.

#### 5.2.2. Exosomal Long Noncoding RNAs (lncRNAs) and Other Nucleic Acids

Exosomes transport not only microRNAs but also long noncoding RNAs (lncRNAs) and other nucleic acids, which play crucial roles in regulating cellular pathways relevant to glaucoma. Examples of lncRNAs identified in aqueous humor-derived exosomes include MALAT1 and HOTAIR, which have been implicated in the modulation of angiogenesis, fibrosis, apoptosis, and immune responses [25,30]. Due to their high disease specificity and stability within exosomes, lncRNAs represent promising emerging biomarkers for both advanced and subclinical ocular pathologies [42]. Profiling these nucleic acids alongside microRNAs allows for a more comprehensive molecular characterization of exosomes, offering valuable insights into glaucoma pathophysiology, including early cellular stress in the trabecular meshwork, ciliary body, and corneal endothelium [25,30].

#### 5.2.3. Exosomal Proteins (Non-Surface Cargo)

Aqueous humor-derived exosomes contain a diverse array of internal proteins and enzymes that reflect the physiological and pathological state of their parent cells. These cargos include enzymes involved in apoptosis and extracellular matrix remodeling (e.g., matrix metalloproteinases, MMPs), oxidative stress-related proteins (e.g., superoxide dismutase, peroxiredoxins), angiogenic factors (e.g., VEGF), complement components (e.g., C3, C5b-9), and inflammatory mediators such as cytokines (e.g., IL-6, TNF-α) [25,30,42]. The composition and relative abundance of these molecules provide crucial insights into trabecular meshwork remodeling, ciliary body dysfunction, and corneal endothelial stress. Profiling exosomal proteins thus offers a powerful approach to elucidate glaucoma pathophysiology and supports the development of novel diagnostic and monitoring strategies [30,42].

#### 5.2.4. Exosomal Lipids

Exosomal lipids constitute another critical component of internal cargo, influencing vesicle biogenesis, membrane stability, and intercellular signaling [30,42]. Common lipid species within exosomes include ceramides, sphingomyelin, phosphatidylserine, and cholesterol. In the aqueous humor, exosomal lipid composition can undergo dynamic changes in response to pathological stimuli, such as oxidative stress, immune activation, barrier dysfunction, and endothelial damage [25]. Alterations in lipid content may modulate inflammatory signaling, membrane fusion events, and overall vesicle function, thereby impacting ocular homeostasis. Profiling these lipid changes provides complementary information to nucleic acid and protein analyses, offering a more comprehensive molecular understanding of glaucoma pathophysiology and identifying potential targets for diagnostic and biosensor-based applications [42].

## 6. Potential Biosensing Strategies for Aqueous Humor–Derived Exosomes in Glaucoma

### 6.1. Scope and Translational Framework of Electrochemical Biosensing for Aqueous Humor Exosomes

Electrochemical biosensors have emerged as powerful platforms to overcome current analytical limitations in the detection of exosomal biomarkers, enabling selective and ultrasensitive analysis and facilitating early-stage disease assessment across a variety of biological matrices, including complex ones, while minimizing matrix-related interferences.

**Figure 1:**
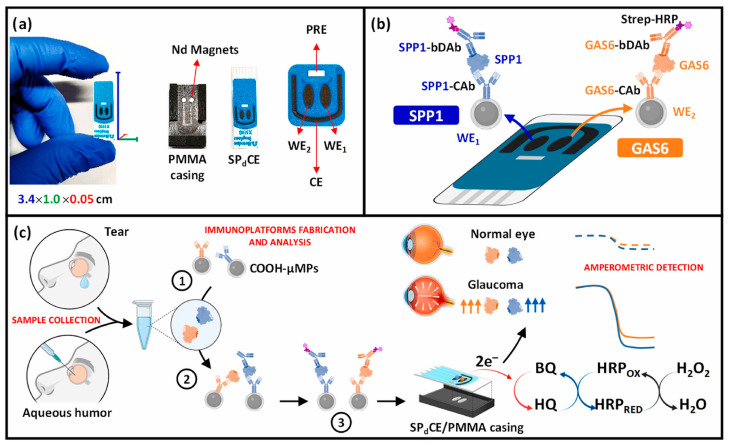
(**a**) Images of the SPdCE dual electrochemical transducers and PMMA housing used to obtain amperometric readings. (**b**) Schematic of the dual SPP1/GAS6 immunoplatform based on sandwich immunoassay formats and enzymatic labeling with the HRP enzyme. (**c**) Immunoreaction and amperometric transduction processes involved in the dual determination of SPP1/GAS6 proteins, and fictitious amperometric responses obtained in the analysis of SPP1 and GAS6 proteins in tear/aqueous humor samples collected from normal and glaucoma eyes. Blue indicates SPP1 and orange indicates GAS6. The dashed line corresponds to the healthy sample, whereas the dotted line corresponds to the diseased sample. Reproduced with permission from Ref. [54].

In the context of ocular biofluids, only a limited number of electrochemical biosensors have been reported for the direct analysis of AH, targeting soluble protein biomarkers rather than exosome-associated species. For instance, in 2023, a microelectrode-based electrochemical sensor was developed for the detection of brain-derived neurotrophic factor (BDNF) in AH, achieving high sensitivity with a low sample consumption of 5 μL [53]. The sensor exhibited a dynamic range of 0.5–50 pg/mL and a detection limit of 0.3 pg/mL and enabled the confirmation of decreased BDNF levels in glaucoma patients. More recently, an electrochemical immunoplatform was reported for the simultaneous detection of two glaucoma-related biomarkers, secreted phosphoprotein 1 (SPP1) and growth arrest–specific 6 (GAS6), using magnetic microparticles and disposable carbon electrodes [54]. The platform was successfully applied to AH, demonstrating high sensitivity, selectivity, and reproducibility, and enabling rapid and multiplexed biomarker analysis, achieving LOD values of 6.0 and 59.0 pg/mL for SPP1 and GAS6, respectively, within only 90 min (Figure 1). Notably, its applicability to tear fluid was also demonstrated as a proof of concept, potentially overcoming the invasiveness associated with ocular sample collection. The results were consistent with ELISA measurements and confirmed significantly elevated biomarker levels in glaucoma patients.

Despite these advances in aqueous humor AH analysis using biosensing platforms, to our knowledge, no electrochemical biosensor has yet been specifically developed or experimentally validated for the detection of aqueous humor–derived exosomes. Accordingly, the following sections critically review electrochemical biosensing studies targeting exosomal surface proteins and intravesicular cargos that have been validated using other biological matrices, such as serum, plasma, or cell culture supernatants, across diverse disease contexts. Only studies addressing biomarkers and molecular pathways relevant to glaucoma pathophysiology have been considered, even when such targets were not investigated in aqueous humor. Rather than constituting direct clinical solutions for AH-derived exosome analysis, these platforms should be regarded as transferable technological frameworks, whose analytical performance, low sample volume requirements, and modular biorecognition architectures render them particularly suitable for future adaptation to AH-derived exosomes. Within this perspective, the analysis of related disease models enables the identification of realistic translational pathways toward the development of next-generation electrochemical biosensors for glaucoma diagnostics.

### 6.2. Electrochemical Detection of Exosomal Surface Proteins: Transferable Platforms for Glaucoma Diagnostics

#### Tetraspanins as Universal Exosome Capture Markers

Most electrochemical biosensing strategies developed to date primarily focus on exosomal surface markers, particularly tetraspanins such as CD63, CD9, and CD81, which serve as robust and widely accepted markers for exosome capture and quantification. These proteins are abundantly expressed on exosomal membranes and provide reliable anchoring points for antibody- or aptamer-based recognition, enabling highly promising analytical performances.

**Figure 2:**
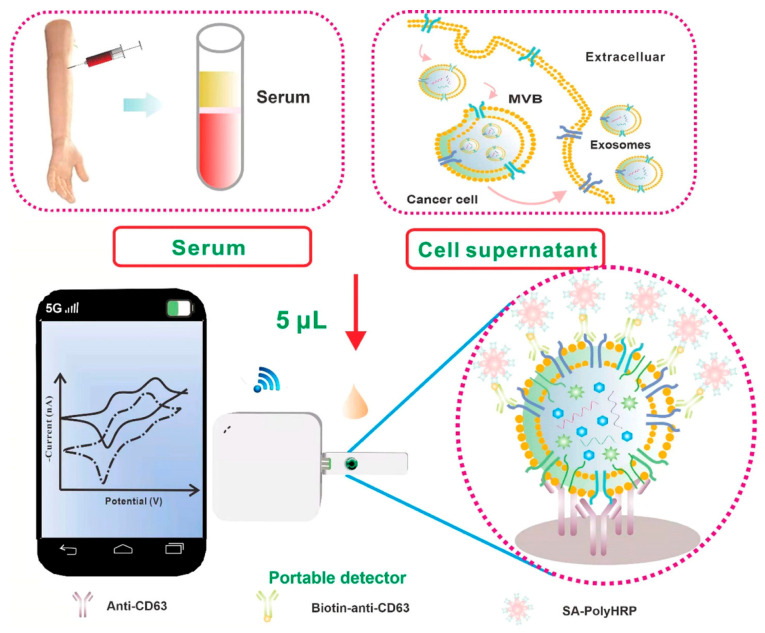
Scheme of the Smartphone-Based Electrochemical Biosensor for Directly Detecting Serum-Derived Exosomes and Monitoring the Secretion of Exosomes. Reproduced with permission from Ref. [55].

Among these, CD63 has been extensively reported as a general exosomal marker across various diseases and is commonly used to confirm the presence of exosomes as biomarkers in early diagnosis and prognosis prediction, particularly in oncology. Su et al. developed a sensitive and portable electrochemical biosensor for the quantitative analysis of exosomes that can be integrated with smartphones for point-of-care applications (Figure 2). Using screen-printed carbon electrodes (SPCEs), this platform enabled the detection of exosome markers in serum and cell culture supernatants in less than two hours using a simplified workflow. Capture antibodies immobilized on SPCEs bound exosomes via hydrophobic interactions, followed by recognition using multiple biotinylated detection antibodies and signal amplification with streptavidin–polyhorseradish peroxidase (SA-polyHRP). This strategy achieved detection of as little as 7.23 ng of exosomes in only 5 μL of sample without additional pretreatment, demonstrating the suitability of SPCE-based platforms for low-volume bioanalysis. The authors further observed reduced levels of CD63-positive exosomes in serum from patients with prostate diseases, consistent with previous observations in human melanoma cells [55].

**Figure 3:**
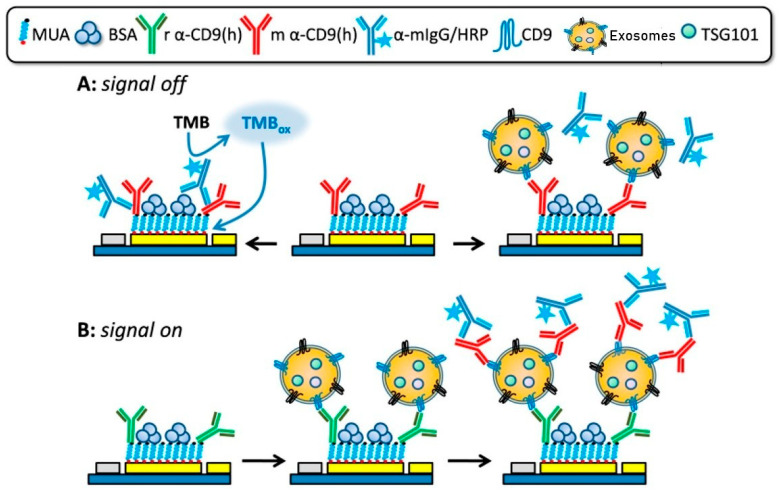
“Signal-Off” and “Signal-On” strategies of an electrochemical sandwich immunosensor for CD9 detection. Reproduced with permission from Ref. [56].

CD9 represents another highly abundant and functionally important tetraspanin involved in membrane organization, vesicle trafficking, and exosome biogenesis. Its stability and accessibility on the exosomal membrane make it an attractive target for electrochemical detection in complex biofluids. Doldán et al. reported a miniaturized electrochemical sandwich immunosensor exploiting CD9 as a surface marker for selective exosome capture and quantification [56]. This platform employs a gold electrode functionalized with anti-CD9 antibodies for primary capture, followed by immune-sandwich formation using a second anti-CD9 antibody and HRP-conjugated secondary antibodies (Figure 3). Both “signal-off” and “signal-on” detection modes were demonstrated, with the latter achieving ultra-low detection limits of approximately 200 exosomes per microliter over a dynamic range spanning nearly four orders of magnitude Notably, the sensor requires only 1.5 μL of sample and exhibits excellent discrimination between exosomes and microvesicles even in complex serum matrices, underscoring its relevance for low-volume clinical samples such as ocular fluids.

**Figure 4:**
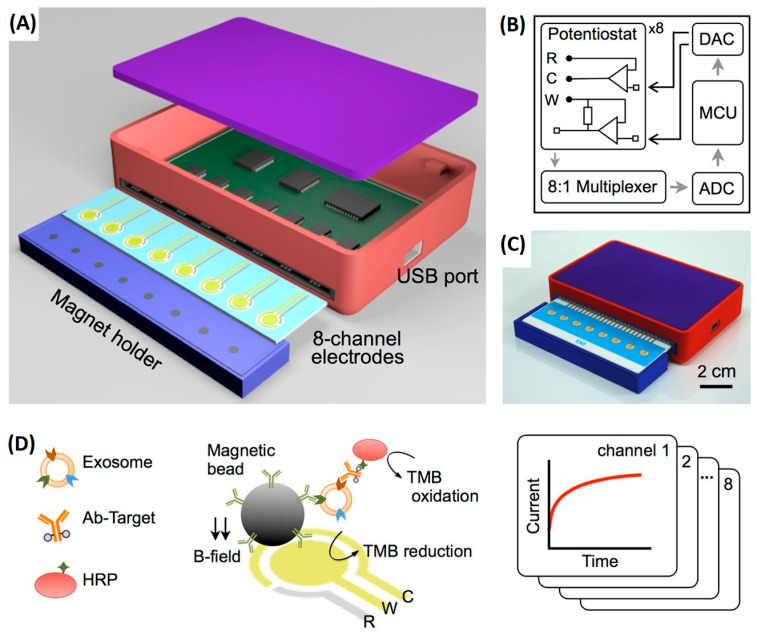
Integrated magnetic–electrochemical exosome (iMEX) platform. (**A**) Sensor schematic showing eight electrodes for simultaneous signal measurement; cylindrical magnets beneath the electrodes concentrate immunomagnetically captured exosomes. (**B**) Circuit diagram of the sensor system, comprising eight potentiostats, an 8-to-1 multiplexer, ADC, DAC, and an MCU; each potentiostat includes reference, counter, and working electrodes. (**C**) Photograph of the packaged device with a compact form factor (9 × 6 × 2 cm^3^). (**D**) Schematic of the iMEX assay, in which exosomes are directly captured from plasma using magnetic beads coated with anti-CD63 antibodies and labelled with HRP for electrochemical detection; gold working and counter electrodes and an Ag/AgCl reference electrode enable simultaneous monitoring of eight channels. Reproduced with permission from Ref. [57].

To overcome the limitations of single-marker analysis, advanced electrochemical platforms capable of multiplexed exosome detection have been developed, enabling simultaneous measurement of multiple surface biomarkers within a single assay and enhancing analytical reliability. In this context, the study reported by Jeong et al. (2016) introduced an integrated magnetic–electrochemical exosome (iMEX) platform capable of detecting multiple exosomal tetraspanins, including CD63, CD9, and CD81 [57]. This system addresses key bottlenecks associated with conventional exosome isolation methods by integrating immunomagnetic capture, enzymatic labeling, and electrochemical signal transduction into a single streamlined workflow. Magnetic microbeads functionalized with antibodies against exosomal surface proteins directly capture extracellular vesicles from unprocessed biological fluids, with CD63 yielding the most robust and reproducible enrichment. HRP-conjugated detection antibodies generate electrochemical signals via TMB-mediated readout, while magnetic concentration of bead-bound vesicles onto microelectrodes enhances sensitivity. The eight-channel portable iMEX device enables quantitative detection from just 10 μL of plasma from ovarian cancer patients, with sensitivity below 10^5^ vesicles and total assay times under one hour, highlighting its strong potential for near-patient and point-of-care applications (Figure 4).

In this context, another multiplexed approach relies on a magneto-mediated electrochemical sensor based on host–guest recognition [58]. In this system, CD63 aptamer-functionalized magnetic beads are used for the selective capture of exosomes, while silica nanoparticles modified with aptamers targeting disease-specific proteins provide a highly specific secondary recognition step. Signal amplification is achieved through the release of a ferrocene-based reporter upon reduction in disulfide bonds, with electrochemical detection carried out on graphene oxide–cucurbit-modified screen-printed carbon electrodes (SPCEs). Overall, these sandwich-type strategies highlight the versatility and adaptability of multiplexed electrochemical platforms for the analysis of glaucoma-relevant exosomal proteins, using CD63 as a stable reference marker for exosome presence.

### 6.3. Electrochemical Detection of Internal Exosomal Nucleic Acids: Focus on miRNAs

Detection of internal exosomal components, particularly microRNAs (miRNAs), is significantly more challenging than surface proteins due to their low abundance within the vesicle lumen. Additional steps, such as vesicle lysis, cargo extraction, or signal amplification, are required, compromising sensor simplicity, reproducibility, and clinical applicability [31,59]. Electrochemical biosensors provide a sensitive and versatile solution to these limitations, enabling selective miRNA detection by combining hybridization-based recognition with powerful signal-amplification approaches, including catalytically driven amplification [60] and enzyme-mediated nucleic acid amplification strategies [61,62]. Indeed, numerous studies have validated the potential of electrochemical devices for miRNA detection in biological fluids such as blood, plasma, and serum [59]. Yet, despite these advances, only a limited number of works have applied electrochemical biosensing directly to miRNAs isolated from exosomes, and, to date, no electrochemical biosensors have been reported for glaucoma-specific exosomal miRNAs. Electrochemical strategies targeting miRNAs are increasingly incorporating signal-amplification chemistries, including hybridization chain reaction, primer exchange reaction, and nanozyme-mediated catalytic enhancement.

**Figure 5:**
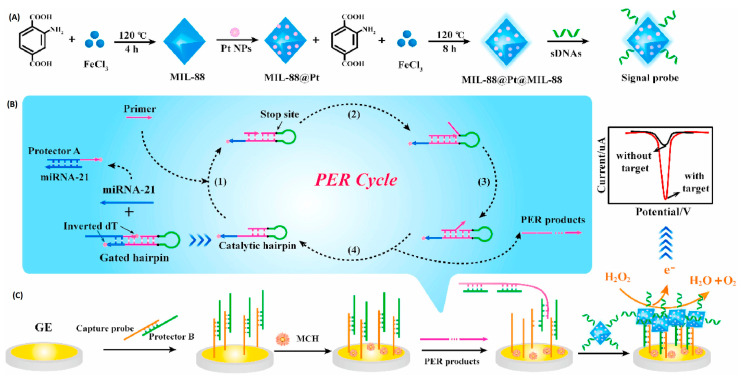
Principle of the electrochemical biosensor based on the cascade PER and MOF@Pt@MOF nanozyme for the detection of exosomal miRNA-21. (**A**) The synthesis route of the MOF@Pt@MOF. (**B**) The operation mechanism of the cascade PER. (**C**) The electrochemical signal produced by MOF@Pt@MOF. Reproduced with permission from Ref. [64].

In this sense, a magnetic bead–assisted approach has been employed to isolate target miRNA-21 from complex matrices, followed by electrochemical readout using redox reporters such as [Fe(CN)_6_]^4−^/^3−^. These systems have achieved detection limits as low as 1.0 pM with high reproducibility in serum and cancer cell samples [63]. More advanced amplification schemes have been reported using cascade primer exchange reactions (PER) coupled with nanozyme-mediated signal enhancement. In one representative platform, PER-generated DNA products enable binding of a multilayered MOF@Pt@MOF nanozyme, which catalyzes hydrogen peroxide decomposition to generate amplified electrochemical signals [64]. This strategy achieved femtomolar-level sensitivity (LOD = 0.29 fM) and single-nucleotide discrimination, successfully detecting exosomal miRNA-21 in tumor cells and patient blood samples with performance comparable to qRT-PCR (Figure 5).

**Figure 6:**
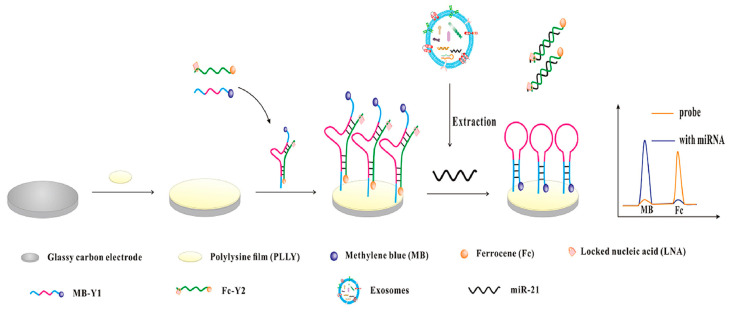
Schematic illustration of the ratiometric electrochemical biosensor for exosomal miR-21 detection. Reproduced with permission from Ref. [66].

To improve reliability, ratiometric electrochemical biosensors provide internal self-calibration through dual-signal readouts. Examples include bipedal DNA walkers driven by LNA-modified strand displacement reactions [65] and amplification-free Y-shaped DNA nanostructures, which convert into hairpins upon target binding, activating methylene blue signals while suppressing ferrocene signals (Figure 6), achieving a detection limit of 2.3 fM [66]. These strategies enhance selectivity and robustness against environmental variability and provide proof-of-concept for adapting miRNA biosensing to glaucoma, where exosomal miRNAs contribute to inflammation, oxidative stress, and extracellular matrix remodeling.

### 6.4. Detection of Internal Exosomal Proteins and Lipids: Current Gaps and Future Opportunities

Proteins represent an important class of internal exosomal biomarkers. While electrochemical biosensors have been successfully developed for the detection of soluble proteins such as matrix metalloproteinases, cytokines, and oxidative stress–related enzymes in diverse biological samples [67,68,69], no electrochemical platforms have yet been specifically designed to detect these targets directly from human exosomes. The transition from soluble protein detection to intravesicular analysis is hindered by several challenges, including the low stoichiometric abundance of specific proteins per vesicle, the requirement for rapid and reproducible membrane lysis, and the technical complexity of integrating multi-step extraction and signal amplification protocols into miniaturized sensor architectures.

Parallel to these protein-related challenges, exosomal lipids, such as ceramides, sphingomyelin, phosphatidylserine, and cholesterol, play fundamental roles in vesicle stability, biogenesis, and intercellular signaling, yet they remain largely unexplored as electrochemical targets. Despite the emergence of novel lipid-recognition strategies and the development of cholesterol-modified probes [57], these approaches are currently in the early stages of development. Significant advancements in sensitivity and selectivity are still required to overcome the current gap between proof-of-concept research and the maturity necessary for clinical translation.

### 6.5. Design Considerations for Next-Generation Biosensors Targeting Aqueous Humor Exosomes

**Figure 7:**
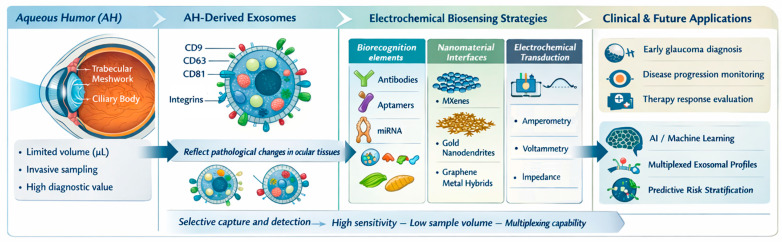
Conceptual summary of electrochemical biosensor designs and exosomal biomarkers relevant to glaucoma, highlighting transferable strategies for future aqueous humor applications. Conceptualization and design by the authors, figure created partially using Biorender.com.

Building upon the diagnostic gaps identified in preceding sections, the evolution of electrochemical platforms reveals a definitive trajectory toward integrated, multiplexed, and clinically deployable architectures. The synergy between high-affinity immunocaptures, isothermal nucleic acid amplification, and nanozyme-mediated catalysis, when coupled with microfluidic integration and ratiometric signal engineering, is significantly expanding the detectable molecular landscape of exosomes while enhancing analytical robustness. Although direct electrochemical detection of internal exosomal proteins and lipids remains an unmet need, addressing these challenges represents a major opportunity for next-generation biosensor design—particularly for glaucoma, where exosome-derived molecular signatures hold growing diagnostic and pathophysiological significance. Figure 7 provides a schematic overview of the most promising electrochemical biosensing strategies, exosomal targets, and translational pathways, highlighting their potential relevance for aqueous humor–derived exosome analysis in glaucoma.

## 7. Future Perspectives

From an analytical perspective, next-generation biosensing platforms must prioritize ultrasensitive detection at microliter or sub-microliter volumes, while minimizing sample handling and preprocessing. Current electrochemical biosensors have demonstrated impressive detection limits for exosomal tetraspanins and selected microRNAs; however, their application to AH-derived exosomes remains unexplored. In the near term, the most clinically realistic advances are expected to arise from microfluidic–electrochemical hybrid platforms capable of integrating on-chip exosome enrichment, concentration, lysis, and multiplexed detection. Systems combining immunomagnetic capture, nanostructured electrodes, and enzyme- or nanozyme-mediated signal amplification offer a particularly promising balance between analytical sensitivity and workflow simplicity, making them well suited to the constraints of AH analysis [56,69]. In parallel, nanomaterial-enhanced signal transduction, using MXenes, gold nanodendrites, or graphene–metal hybrids, represents a powerful strategy to further boost sensitivity while preserving electrode miniaturization and clinical compatibility [70].

From a biological standpoint, expanding the repertoire of glaucoma-relevant exosomal biomarkers represents a critical priority. While most current studies emphasize canonical tetraspanins, AH-derived exosomes harbor a far richer molecular landscape. Future efforts should focus on systematic multi-omic profiling of AH exosomes using proteomics, lipidomics, and small-RNA sequencing to identify biomarker signatures mechanistically linked to glaucomatous pathology [30]. Particular attention should be paid to pathways associated with trabecular meshwork dysfunction, oxidative stress, mitochondrial impairment, glial activation, and ferroptosis—processes increasingly recognized as central to glaucomatous neurodegeneration [8,71]. Biomarkers reflecting these mechanisms may enable earlier disease stratification and more precise monitoring of progression than pressure-based metrics alone.

A major analytical bottleneck remains the detection of internal exosomal cargo, which is physically shielded within the vesicle lumen and is typically present at extremely low abundance. Exosomal miRNAs already show strong promise as early glaucoma biomarkers, but their reliable detection requires advanced amplification strategies. DNA nanotechnology, catalytic hairpin assembly, primer exchange reactions, and nanozyme-mediated catalysis represent particularly compelling directions for future biosensor development [59,62]. Beyond nucleic acids, internal proteins, enzymatic activities, and lipid signatures may provide complementary mechanistic insights. Progress in this area will likely depend on the development of lysis-integrated or membrane-disruption-assisted electrochemical platforms capable of accessing intravesicular content without compromising assay robustness or reproducibility.

From a translational perspective, the most significant challenge remains clinical feasibility. Ethical and practical considerations limit AH collection to microliter volumes and to specific clinical scenarios, such as cataract surgery or anterior chamber paracentesis. Consequently, future biosensors must operate reliably with minimal sample volumes, demonstrate high reproducibility across operators and settings, and deliver rapid readouts compatible with near-patient or point-of-care use. While direct AH analysis is likely to remain restricted to selected contexts, alternative strategies, such as tear-based or implantable biosensing systems that indirectly reflect AH molecular signatures, may offer longer-term solutions for longitudinal disease monitoring [72]. In parallel, regulatory considerations, including standardization of extracellular vesicle isolation protocols, calibration strategies, and large-cohort clinical validation, will be essential for eventual clinical translation.

Looking forward, the integration of AH-exosome biosensing with AI-driven diagnostic frameworks represents a particularly transformative opportunity. Machine learning models trained on multiplexed exosomal protein and RNA profiles could identify subtle biomarker patterns indicative of early glaucomatous changes, potentially surpassing the diagnostic sensitivity of current structural or intraocular pressure–based assessments [30]. Such integrative approaches may ultimately enable personalized glaucoma management, supporting dynamic monitoring of neurodegenerative progression and therapeutic response.

In summary, although significant challenges remain, the convergence of advanced electrochemical biosensing technologies, multi-omic exosome characterization, and computational analytics positions exosome-based diagnostics at the forefront of next-generation glaucoma research. Continued innovation across these domains has the potential to fundamentally reshape early detection strategies, improve disease monitoring, and support individualized treatment paradigms in glaucoma care.

## Data Availability

The original contributions presented in the study are included in the article; further inquiries can be directed to the corresponding author.
